# RNASET2 tag SNP but not CCR6 polymorphisms is associated with autoimmune thyroid diseases in the Chinese Han population

**DOI:** 10.1186/s12881-015-0150-9

**Published:** 2015-02-26

**Authors:** Xiao-jun Chen, Xiao-hua Gong, Ni Yan, Shuai Meng, Qiu Qin, Yan-Fei Jiang, Hai-Yan Zheng, Jin-an Zhang

**Affiliations:** Department of Endocrinology, The First Affiliated Hospital of Wenzhou Medical University, No.2 Fuxuexiang Road, Ouhai District, 325000 Wenzhou, China; Department of Endocrinology, Jinshan Hospital of Fudan University, No.1508 Longhang Road, Jinshan District, 201508 Shanghai, China

**Keywords:** RNASET2, CCR6, Single nucleotide polymorphisms (SNPs), Autoimmune thyroid diseases (AITDs), Graves’ disease (GD), Hashimoto’s thyroiditis (HT)

## Abstract

**Background:**

Polymorphisms of the CC chemokine receptor 6 (CCR6) and RNASET2 tag SNP have been shown to be associated with the susceptibility to several immune-related diseases. This study was conducted to identify the association of CCR6 and RNASET2 tag SNP with autoimmune thyroid diseases (AITDs) in the Chinese Han population.

**Methods:**

We enrolled 1061 patients with AITDs, including 701 patients with Graves’ disease (GD) and 360 patients with Hashimoto’s thyroiditis (HT), and 938 healthy individuals for a case–control genetic association study. Three CCR6 single nucleotides polymorphisms (SNPs) (rs3093023/rs3093024/rs6902119) and one tagging SNP (rs9355610) within RNASET2 gene were selected for genotyping by multiplex polymerase chain reaction (PCR) and ligase detection reaction (LDR).

**Results:**

The frequency of rs9355610 genotypes in the patients with GD differed significantly from that in the controls (p = 0.017). The frequency of the minor G allele of rs9355610 was significantly higher in the GD patients than in the healthy controls (p = 0.005, OR = 1.225, 95% CI:1.063-1.412). However, we could not find significant differences in the genotype or allele frequencies of HT patients compared with healthy controls. After gender stratification, the frequency of the minor G allele in both male and female GD patients was significantly higher than that in the healthy controls (p = 0.036, OR = 1.308, 95% CI:1.017-1.684 ; p = 0.048, OR = 1.19, 95% CI:1.001-1.413; respectively);. Furthermore, the frequency of haplotype AT in GD patients was significantly lower than that in their control groups (p = 0.003) and showed a protective effect against GD (OR = 0.806, 95% CI: 0.699-0.929). The frequency of haplotype GT in GD patients was significantly higher than that in their control groups (p = 0.048), indicating that GT was the risk haplotype to GD (OR = 1.267, 95% CI: 1.001-1.603). There were no significant differences in the allele or genotype frequencies of three SNPs of CCR6 (rs3093023/rs3093024/ rs6902119) gene between GD patients, HT patients and controls.

**Conclusions:**

Our results suggest that the rs9355610 tag SNP of RNASET2 gene is positively associated with susceptibility to GD in the Chinese Han population. No association was found for the tested CCR6 SNPs.

## Background

Autoimmune thyroid disease (AITD) is an organ-specific autoimmune disease characterized by the break-down of self-tolerance to thyroid antigens and the occurrence in the serum of antibodies against thyroid self-antigens. Two typical AITDs, Graves’ disease (GD) and Hashimoto’s thyroiditis (HT), affect up to 5% of the general population [1], and both of are characterized pathologically by infiltration of the thyroid by T and B-cells reactive to thyroid antigens, biochemically by the production of thyroid autoantibodies, and clinically by abnormal thyroid functions (hyperthyroidism in GD and hypothyroidism in HT) [2].

The current paradigm is that AITDs are complex diseases in which susceptibility genes and environmental triggers act in concert to initiate the autoimmune response to the thyroid. While the exact etiology of AITD is incompletely known, there is abundant evidence for a strong genetic influence on the development of AITD. The putative GD and HT susceptibility genes include both immune modifying genes HLA Class II gene [3,4], cytotoxic T lymphocyte-associated factor 4 (CTLA-4) gene [5,6], CD40 gene [5-7], protein tyrosine phosphatase-22 (PTPN22) gene [5], and other thyroid-specific genes for the thyroid stimulating hormone receptor (TSHR) [5,8] and thyroglobulin (TG) [9], as well as the novel proinflammatory cytokine such as interleukin-17 (IL-17) [10] and IL-21 [11]. All these data suggest that genetic factors may play an important role in the pathogenesis of AITD.

CC chemokine receptor 6 (CCR6), a member of the β chemokine receptor family, is expressed in immature dendritic cells, memory T cells, lymphocytes and B cells [12-14]. The CCR6 gene encodes C-C motif chemokine receptor 6, the receptor for CCL20 and a surface marker for IL-17–producing T helper 17 (Th17) cells [13]. It is believed that CCL20/CCR6 signaling plays a role in the recruitment of not only immature dendritic cells and their precursors, but also Th17 cells, into sites of potential antigen entry [13]. The pathogenesis of various autoimmune diseases and inflammatory diseases has been linked to the involvement of Th17 cells, most of them reporting CCR6 as a key factor for involvement of Th17 cells [15-17]. We also have reported that IL-17 might play an key pole in the pathogenesis of HT and IL-17A/F polymorphisms may affect the susceptibility to AITD [10,18]. Recent studies have suggested that CCR6 polymorphisms are associated with an increased risk for a variety of autoimmune- or immune-mediated diseases, such as rheumatoid arthritis (RA) [19,20], Crohn’s disease [21], systemic sclerosis (SSc) [22] and vitiligo [23]. However, it remains unknown whether CCR6 polymorphisms are associated with AITD in the Chinese population which was the subject of this study.

Chu et al. carried out a genome-wide association study (GWAS) of individuals with GD in a Chinese Han population and identified novel GD susceptibility loci rs9355610, within 6q27 chromosomal bands that contribute to generalized GD risk [24]. Of interest is the fact that HT is also the clinical subtype of AITDs and shares the similar genetic predisposition with GD. Whether genes in this region are also associated with HT is unknown and is therefore the subject of this study.

In this study, we therefore conducted a large case–control study to determine the genetic association of the selected SNP of CCR6(rs3093023/rs3093024/rs6902119) and one tagging SNP (rs9355610) within RNASET2 with AITD in a cohort of the Chinese Han population. Furthermore, we analyzed the association between genotypes and AITD clinical characteristics.

## Methods

### Study population

The study population (n = 1999) consisted of 1061 Chinese Han non-related AITD patients including 701 patients with GD and 360 patients with HT; and 938 unrelated, ethnically matched healthy controls. All AITDs patients were enrolled from the Outpatient Department and Inpatient Department of Endocrinology of Jinshan Hospital of Fudan University; ethnically and geographically matched healthy controls were recruited from the Health Check-Up Center of the hospital. The subjects were all of self-reported Chinese Han ethnicity. This study was approved by the Ethics Committee of Jinshan Hospital of Fudan University. Written informed consent was obtained from all subjects. All procedures followed the tenets of the Declaration of Helsinki.

Inclusion criteria: AITDs patients were diagnosed as described in our published papers [25]. GD was diagnosed based on clinical and laboratory biochemical evidence of hyperthyroidism by the presence of diffuse goiter, and supported by the positive anti-thyroid stimulating hormone receptor antibody (TRAb) and/or anti-thyroid peroxidase antibody(TPO-Ab) and/or anti-thyroglobulin(Tg-Ab) and/or exophthalmos. HT was diagnosed based on the presence of an enlarged thyroid and either TPO-Ab or Tg-Ab, with or without documented clinical and biochemical hypothyroidism. For the suspicious cases of HT, diagnoses were confirmed by fine needle aspiration biopsies (FNAB).

Exclusion criteria: All healthy subjects without clinical evidence (goiter, TPOAb, and TGAb) and family history of thyroid diseases and any other autoimmune diseases.

The clinical manifestations of AITD patients include: (1) adult- or childhood- onset AITDs, according to the age of disease onset (≤18 years versus ≥19 years); (2) presence or absence of ophthalmopathy in GD group (defined as a distinctive disorder characterized by inflammation and swelling of the extraocular muscles, eyelid retraction, periorbital edema, episcleral vascular injection, conjunctive swelling and proptosis); (3) presence or absence of thyroid dysfunction in HT group: euthyroid status or hypothyroidism; and (4) presence or absence of AITDs family history.

### DNA extraction and genotyping

From all the study participants, peripheral venous blood of 2 mL was collected and genomic DNA was extracted from peripheral blood cells using the Nucleon Bacc kit (TianGen Biotech Co., Ltd., Beijing, China), according to the manufactures’ guidelines. Four selected SNPs (rs3093023/rs3093024/rs6902119/rs9355610) were genotyped by ligase detection reactions (LDR) platform in Shanghai Biowing Applied Biotechnology Company (http://www.biowing.com.cn/). This is a well-established platform used by many researchers for studies including SNP genotyping [25]. The target DNA sequences were amplified using multiplex polymerase chain reaction (PCR) method with specific primer sequences as follows:

- rs3093023 forward: 5'-CACCTCACAGTGTCTATGC-3'
- reverse: 5'-GATCCTCTTAGATCTCACTC-3';
- rs3093024 forward: 5'- TGATATGCAGAGAGCCTACG-3'
- reverse: 5'- TCTGTGCTCCCTCCTGTGAA-3';
- rs6902119 forward: 5'- CATCAAAGCAACAGTCAGTC-3'
- reverse: 5'- TGTGGAGAAAGAATCCAAGC-3';
- rs9355610 forward: 5'- ATGAAAGGTTAGCACACCAG-3'
- reverse: 5'- TGGCGCATGGCTCAGAATAC-3'.

Extracted DNA was amplified by PCR using the GeneAmp PCR System 9600 (Perkin Elmer, Waltham, MA, USA) for PCR amplification. The reaction course included initial denaturation at 95°C for 2 min, denaturation at 94°C for 30 s, annealing at 56°C for 1 min, extension at 65°C for 30 s with 35 cycles, and final extension at 65°C for 10 min. After the reaction, 2μL of each product was run in a 3.0% agarose gel to determine whether the reaction had been successful. After the completion of the amplification, the ligation detection reaction for each subject was carried out in a final volume of 10 μl containing 1 μL buffer (1×), 1 μL Prob mix (2 pmol/μL), 1 μL of Multi-PCR product (100 ng/μL), 6.95 μL H_2_O, 0.05 μL of 2 U/μL Taq DNA ligase (New England Biolabs, Ipswich, MA, USA). The LDR was performed using 40 cycles of denaturation at 95°C for 2 min, annealing at 94°C for 15 s and extension at 50°C for 25 s. The LDR fluorescent product was analyzed using an ABI sequencer 377 (Applied Biosystems), and the results were analyzed with Genemapper software.

In addition to ensuring quality of the assay, we made double positive controls (duplication of the same DNA samples) and negative controls (blank samples without DNA) in the processor of SNP genotyping. Quality control analysis was also performed so that only SNPs and samples which had passed the 95% quality control threshold were subjected to further statistical analysis.

### Statistical analysis

Statistical analyses were performed using SPSS version 17.0 software (SPSS Inc., Chicago, IL, USA). Each genetic marker was tested for Hardy-Weinberg equilibrium in the control population using the HWE program (http://ihg.gsf.de/cgi-bin/hw/hwa1.pl). A linkage disequilibrium (LD) map used to define the haplotype blocks was constructed using Haploview software (version 4.2; http://www.broadinstitute.org/haploview/haploview). The haplotype analysis was performed to compare distributions of haplotype frequencies of SNPs between cases and controls. All the allele and genotype frequencies between the patients and the controls were assessed by χ^2^-test. The two-tailed independent samples t test was performed to evaluate the diversity of parameters between groups of different genotypes. To examine whether each SNP independently contributes to susceptibility to AITD, conditional logistic regression analysis was employed. All tests were two-tailed, considering p-values <0.05 as significant. Odds ratios were calculated for the minor allele at each SNP.

### Power calculation

The power of the data was assessed using the Power and Sample Size Calculation software (Ps; version 3.0; http://biostat.mc.vanderbilt.edu) [26]. Taking into account the expected frequency of the CCR6 rs9355610 minor allele (46.1%) in the general population, the combined set of 1061 AITD cases and 938 controls provided a power of 81.7% to detect an association between AITD and these variants, with an OR of 1.2 at the 5% significance level.

## Results

### Clinical characteristics of the subjects

To investigate whether four selected SNPs contribute to the susceptibility to AITD, we performed a case and control association study. As shown in Table 1, in the GD patients 69.5% were females, the mean age was 36.86 years; in the HT patients 87.8% were females, the mean age was 35.09 years. In the healthy controls, 67.1% individuals were females and the overall mean age was 38.61 years. There was no difference in age distribution between GD, HT patients and controls. The clinical findings of AITD patients are also presented in Table 1.

**Table 1:** **The demographic and clinical characteristics of the AITDs patients and the controls**

**Characteristics**	**GD patients (n = 701 )**	**HT patients (n = 360)**	**Controls (n = 938)**
Gender			
Male (%)	214(30.5)	44(12.2)	309(32.9)
Female (%)	487(69.5)	316(87.8)	629(67.1)
Age (mean ± SD)	36.86 ± 14.549	35.09 ± 13.786	38.61 ± 9.202
Age of onset	33.98 ± 14.344	32.79 ± 13.465	-
≤18 years	113(16.12)	51(14.2)	-
≥19 years	588(83.88)	309(85.8)	-
Thyroid size			-
Normal size (%)	22(3.1)	12(3.3)	-
Degree I (%)	127(18.1)	59(16.4)	-
Degree II (%)	425(60.7)	253(70.3)	-
Degree III (%)	127(18.1)	36(10.0)	-
Family history (%)	174(24.8)	79(21.9)	
Ophthalmopathy (%)	135(19.3)	9(2.5)	-
Thyroid antibodies			-
TPOAb	398(56.7)	252(70.0)	-
TgAb	303(43.2)	226(62.8)	-

### Association of rs3093023/rs3093024/rs6902119/rs9355610 polymorphisms with susceptibility to AITD

The distribution of genotype frequencies of each SNP in both the patients and the controls were in Hardy-Weinberg equilibrium (p > 0.05).

Differences in genotypic and allelic frequencies of SNPs between the patients and the controls were compared and are presented in Table 2. There were no significant differences in the allele or genotype frequencies of three SNPs of CCR6 (rs3093023, rs3093024, rs6902119) between AITD patients, GD patients, HT patients and controls. However, rs9355610 appeared to be associated with AITD susceptibility. The frequencies of rs9355610 genotypes in patients with GD (GG, 25.3%; AG, 51.7% and AA, 23.0%) differed significantly from those in the controls (GG, 30.0%; AG, 50.3% and AA, 19.7%) (p = 0.017). We observed a significant increase in the frequency of the minor G allele of rs9355610 in the AITD and GD patients compared with that in healthy controls and seemed to have higher risks for AITD and GD (in AITD: 49.9% vs.46.1%; p = 0.018, OR = 1.166, 95% CI = 1.026-1.324; in GD:51.2% vs. 46.1%; p = 0.005, OR = 1.225, 95% CI = 1.063-1.412). However, we could not find significant differences in the genotype or allele frequencies of HT patients compared with those of healthy controls.

**Table 2:** **Genotype and allele distribution of CCR 6- RNASET2 gene in AITDs patients, GD patients, HT patients, and controls**

**SNP**	**Allele/**			**P**_**a**_		**P**_**b**_		**P**_**c**_
	**Genotype**	**Controls**	**AITD**	**(OR, 95% CI)**	**GD**	**(OR, 95% CI)**	**HT**	**(OR, 95% CI)**
rs3093023	GG	329(36.9)	341(33.4)	0.252	224(33.3)	0.242	117(33.4)	0.428
	AG	423(47.5)	507(49.6)		327(48.7)		180(51.4)	
	AA	139(15.6)	174(17.0)		121(18.0)		53(15.2)	
	A	701(39.3)	855(41.8)	0.118	569(42.3)	0.091	286(40.9)	0.486
	G	1081(60.7)	1189(58.2)		775(57.7)		414(59.1)	
rs3093024	GG	328(36.9)	341(33.4)	0.264	224(33.4)	0.253	117(33.4)	0.430
	AG	422(47.5)	506(49.6)		326(48.6)		180(51.4)	
	AA	139(15.6)	174(17.0)		121(18.0)		53(15.2)	
	A	700(39.4)	854(41.8)	0.124	568(42.3)	0.096	286(40.9)	0496
	G	1078(60.6)	1188(58.2)		774(57.7)		414(59.1)	
rs6902119	TT	344(38.7)	373(36.5)	0.468	233(34.7)	0.183	140(40.0)	0.888
	CT	420(47.2)	487(47.7)		327(48.7)		160(45.7)	
	CC	126(14.1)	162(15.8)		112(16.6)		50(14.3)	
	C	672(37.8)	811(39.7)	0.223	551(41.0)	0.066	260(37.1)	0.778
	T	1108(62.2)	1233(60.3)		793(59.0)		440(62.9)	
rs9355610	GG	187(30.0)	248(24.2)	0.058	169(25.3)	0.017	79(22.6)	0.799
	AG	448(50.3)	520(51.1)		345(51.7)		175(50.0)	
	AA	256(19.7)	249(24.7)		153(23.0)		96(27.4)	
	G	822(46.1)	1016(49.9)	0.018	683(51.2)	0.005	333(47.6)	0.516
				1.166(1.026-1.324)		1.225(1.063-1.412)		
	A	960(53.9)	1018(50.1)		651(48.8)		367(52.4)	

P_a_ value, AITD versus Controls; P_b_ value, GD versus Controls; P_c_ value, HT versus Controls; OR, odd ratio; 95% CI, 95% confidence intervals.

After gender stratification (shown in Table 3), the frequency of the minor G allele of rs9355610 in both male and female GD patients was significantly higher than that in the healthy controls (p = 0.036, 0.048; respectively); but the degree of risk for GD was higher in male group than in female group (OR =1.308, 95% CI: 1.017-1.684 in males; OR = 1.19,95% CI = 1.001-1.413 in females); the genotype and the minor G allele distribution of rs9355610 did not reveal any significant association with HT.

**Table 3:** **Analysis of genotype and allele distribution of rs9355610 SNP in the GD patients and the controls after gender stratification**

**SNP**	**Alleles/**	**Controls**		**GD**		**HT**		**P**_**a**_		**P**_**b**_	
								**OR (95% CI)**		**OR (95% CI)**	
	**Genotypes**	**M (n = 298)**	**F (n = 593)**	**M (n = 205)**	**F (n = 462)**	**M** **(n = 43)**	**F** **(n = 307)**	**M**	**F**	**M**	**F**
rs9355610	GG	63(21.1)	124(20.9)	56(27.3)	113(24.5)	8(18.6)	71(23.1)	0.110	0.124	0.741	0.675
	AG	148(49.7)	300(50.6)	104(50.7)	241(52.2)	20(46.5)	155(50.5)				
	AA	87(29.2)	169(28.5)	45(22.0)	108(23.3)	15(34.9)	81(26.4)				
	G	274(45.9)	548(46.2)	216(52.7)	467(50.5)	36(41.9)	297(48.4)	0.036	0.048	0.474	0.383
								1.308	1.19		
	A	322(54.1)	638(53.8)	194(47.3)	457(49.5)	50(58.1)	317(51.6)	1.017-1.684	1.001-1.413	-	-

M, male; F, female; P_a_ value, GD versus Controls; P_b_ value, HT versus Controls; OR, odd ratio; 95% CI, 95% confidence intervals.

To examine the contribution of each SNP to susceptibility to AITD, conditional logistic regression analysis was conducted. As shown in Table 4, the association of rs9355610 remained significant after adjustment for rs6902119 SNP genotypes. Adjusted P values (P adjusted) for rs9355610 under the dominant model were 0.024 after adjustment for rs6902119. The results suggest that rs9355610 SNP is independently associated with GD.

**Table 4:** Interaction analysis of gene-gene in AITD, GD and HT patients, by logistic regression*

**Codominant model**									
**SNP**	**rs3093024**			**rs6902119**			**rs9355610**		
rs3093023	0.970	0.998	0.578	0.842	0.886	0.447	0.821	0.353	0.391
rs3093024									
rs6902119							0.941	0.492	0.441
Dominant model									
SNP									
rs3093023	0.392	0.721	0.106	1.0	1.0	0.09	0.738	0.874	0.157
rs3093024					1.0				
rs6902119							0.370	0.024	0.181
Recessive									
SNP									
rs3093023	0.668	0.966	0.191	0.514	0.287	0.999	0.391	0.432	0.101
rs3093024						0.999			
rs6902119							0.679	0.937	0.769

*The data are presented as P_adjusted_ values (AITD/ GD/ HT) from a multiplicative interaction model. The regression models were adjusted for age and sex.

### Haplotype analysis

**Figure 1:**
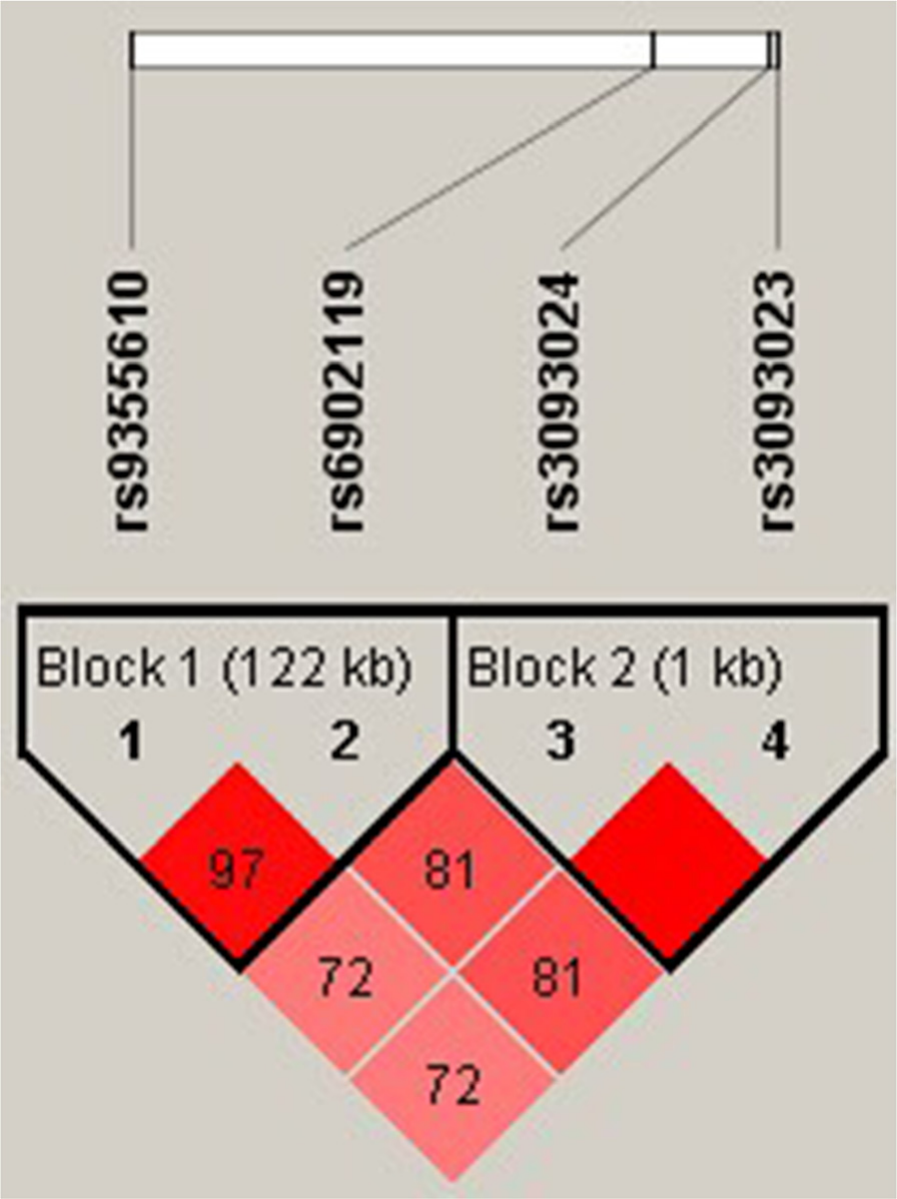
**Linkage disequilibrium (LD) block defined by the Haploview 4.2.**

Linkage analysis of the four tested SNPs in the controls showed moderate to strong linkage disequilibrium (Table 5). To further identify whether haplotypes of those 4 SNPs were correlated with AITD, we created an LD map and analyzed haplotype frequency differences between the AITD patients and the controls. Two detected LD blocks shown in Figure 1 are rs9355610-rs6902119, rs3093023-rs3093024. Table 6 shows the frequency of haplotype AT in AITD and GD patients were significantly lower than that in their control groups (p = 0.016, 0.003),the AT haplotype showed a protective effect on AITD and GD (OR = 0.856,95% CI = 0.754-0.972; OR = 0.806, 95% CI = 0.699-0.929); the frequency of haplotype GT in AITD and GD patients were significantly higher than that in their control groups (p = 0.045, 0.048; respectively), indicating that GT was the risk haplotype to AITD and GD (OR = 1.245, 95% CI = 1.005-1.541; OR = 1.267, 95% CI = 1.001-1.603). However, the haplotypes of block 2 were neither associated with GD nor with HT.

**Table 5:** **Linkage disequilibrium in controls**

**L1**	**L2**	**D'**	**r^2**
		**Controls**	**Controls**
rs9355610	rs6902119	0.97	0.668
rs9355610	rs3093024	0.727	0.402
rs9355610	rs3093023	0.724	0.400
rs6902119	rs3093024	0.819	0.625
rs6902119	rs3093023	0.817	0.621
rs3093024	rs3093023	1.000	0.998

**Table 6:** **Haplotype analysis of AITDs patients and controls**

**Haplotype**	**Controls (Frequency)**	**AITD**	**P**	**OR (95% CI)**	**GD (Frequency)**	**P**	**OR (95% CI)**	**HT (Frequency)**	**P**	**OR (95% CI)**
Block 1										
AT	952(53.3)	1010(49.4)	0.016	0.856	644(47.9)	0.003	0.806 (0.699-0.929)	366(52.2)	0.626	-
				0.754-0.972						
GC	663(37.2)	795(38.9)	0.265	-	536(39.9)	0.116	-	259(37.0)	0.955	-
GT	160(8.9)	223(11.0)	0.045	1.245	149(11.1)	0.048	1.267 (1.001-1.603)	74(10.6)	0.215	-
				1.005-1.541						
Block 2										
GG	1079(60.5)	1188(58.2)	0.136		774(57.7)	0.105	-	414(59.1)	0.519	-
AA	703(39.5)	854(41.8)	0.136		568(42.3)	0.105	-	286(40.9)	0.519	-

AITDs, autoimmune thyroid diseases; GD, Graves’ disease; HT, Hashimoto’s thyroiditis; OR, odds ratio; CI, confidence interval.

### Genotype and clinical phenotype correlations

We also conducted a study on the effects of clinical parameters, including family history, age of onset (≤18 years or ≥ 19 years), ophthalmopathy in GD patients, intractable GD or GD in remission and thyroid dysfunction in HT patients (Hypothyroidism or normal), on the association of the tested four SNPs with AITD. Unfortunately, the results demonstrated no detectable association of these parameters with these SNPs (data not shown).

## Discussion

CCR6 encodes a receptor for macrophage inflammatory protein-3a (CCL20) that is expressed in unactivated memory B and T cells, Th17 cells and some dendritic cells and that plays a key role in B-cell differentiation and tissue-specific migration of dendritic and T cells during inflammatory and immunological responses [27]. A number of autoimmune diseases may share similar gene susceptibility and previous studies have reported significant associations between genetic polymorphisms of CCR6 and certain autoimmune diseases such as RA, Crohn’s disease, SSc and vitiligo [19-23]. In this study, we investigated whether polymorphisms of CCR6 including rs3093023, rs3093024, rs6902119 and the RNASET2 tagSNP rs9355610 contributed to development of AITD in Chinese Han case–control cohorts. To our knowledge, the present study, which analyzed data from a large population of Chinese Han subjects, was the first to demonstrate an association between CCR6 polymorphisms and susceptibility to AITD in the Chinese Han population. Our results showed no association of three SNPs out of the four tested SNPs, i.e. rs3093023, rs3093024 and rs6902119 in the CCR6 gene, with AITD; however, the minor G allele of rs9355610 was significantly associated with susceptibility to GD.

With regard to the selection of the three SNPs of CCR6, the strategy was principally based on the papers reported recently, which are mainly about CCR6 polymorphisms with RA and other autoimmune diseases using candidate gene approaches and genome-wide association studies (GWAS) [19,20]. Recent GWAS have identified SNPs in the 6q27 region associated with RA (rs3093024 and rs3093023 near CCR6) [19,20] and vitiligo (rs6902119 near CCR6) [28]. Rs3093023/rs3093024 have been proved to be as the new risk locis for RA in different ethnic groups, including Japanese [20], Singaporeans [29] and Europeans [19]; rs6902119 has been identified as risk loci for vitiligo in Japanese [28]. Unexpectedly, researchers did not detect any significant associations in the Chinese Han population between rs3093023/rs3093024/rs6902119 and Vogt-Koyanagi-Harada Syndrome, a well known autoimmune disease involving the eye, the neurologic/auditory and integument organs [30]. In the association study on CCR6 SNPs (rs3093023/rs3093024/rs6902119) with susceptibility to Behcet’s disease, no association was found in the Chinese Han population, either [31]. To our knowledge, there has been no association study on CCR6 SNPs (rs3093023/rs3093024/rs6902119) with AITD in the Chinese population. Our present study showed no significant associations between three tested SNPs of CCR6 and AITD patients in the Chinese population (p > 0.05), which was consistent with that of the above association study reported in Vogt-Koyanagi-Harada Syndrome and Behcet’s disease in the Chinese han population. The absence of an association in our study is contrary to the results reported in other autoimmune diseases including RA, SSc and vitiligo in European and Asian populations [19-23,28,29]. This might be explained by the fact that the etiology and pathogenesis of AITD is different from that of the other autoimmune diseases. Another possibility could be that Chinese Han people have a genetic background different from that of other ethnic populations.

Recently Chu et al. [24] has conducted a two-stage GWAS within the Chinese Han population and identified rs9355610 as the novel GD susceptibility locus within 6q27 chromosomal near CCR6 gene. The rs9355610 is located in a block spanning the 5’ exons of RNASET2 and the intergenic region in the Chinese population. It is found that the risk allele G of rs9355610 is significantly associated with reduced mRNA level of RNASET2 only in CD4^+^ and CD8^+^ T cells isolated from 106 healthy Chinese (p = 6.42 × 10^−11^and p = 2.00 × 10^−7^, respectively) [24]. RNASET2 is the only RNase T2 family member in humans; the study showed that omega-1, an RNase T2 family member secreted from the eggs of Schistosoma mansoni, plays a role in priming human dendritic cells for Th2 polarization of CD4^+^ T cells [32], indicating that human RNASET2 might be involved in immune response and therefore underlie the pathogenesis of GD.

A study carried out in the Polish Caucasian population with 560 GD patients and 1475 unrelated healthy controls using TaqMan assay showed no significant association of allelic frequency distribution of rs9355610 between GD and controls (p = 0.082) [33]. In our present study, we found that the G allele of rs9355610 significantly increased the risk of GD (p = 0.005, OR = 1.225), but did not associate with HT. This result is consistent with that of the report on 457 Japanese AITD patients and 222 matched healthy controls which found that the SNPs rs9355610 was significantly associated with GD (p = 0.047), the G allele of rs9355610 was higher in GD patients than in controls (p =0.0055, OR = 1.42), and the GG genotype was also significantly increased in GD patients compared with that in the controls (p = 0.026, OR = 1.63). All these evidently indicated that the TT genotype could increase susceptibility to GD; nevertheless, there was no association between rs9355610 and HT [34].

Because analysis of haplotype provides the genetic information of multiple SNPs, this is a powerful method for the identification of genes contributing to complex diseases. In our study, the associated haplotype was in the same LD block. The rate of AT haplotype was lower in GD, suggesting a protective effect on GD, while the rate of the haplotype GT was higher, indicating a significant risk factor for GT. This consistency with allele analysis strongly confirms that G allele of rs9355610 was a predisposing factor for AITD.

It is worthwhile to point out that several limitations exist in our study. Although

The association with the RNASET2 gene suggests that G allele affect expression of RNASET2 gene in CD4+ and CD8^+^ T cell, we did not perform biological function tests in control carriers of the various phenotypes. Because of limited SNPs included in our study, future study using the high-throughput analytic methods available (i.e., gene sequencing) may help clarify our findings and provide further insight into the role of CCR6 in AITD.

## Conclusions

This study indicates that the G allele of the RNASET2 tag SNP rs9355610, but not rs3093023, rs3093024 or rs6902119 in the CCR6 gene, is associated with susceptibility to GD in the Chinese Han population.

## Abbreviations

CCR6: CC chemokine receptor 6
AITDs: Autoimmune thyroid diseases
GD: Graves’ disease
HT: Hashimoto’s thyroiditis
SNPs: Single nucleotides polymorphisms
PCR: Polymerase chain reaction
LDR: Ligase detection reaction
CTLA-4: Cytotoxic T lymphocyte-associated factor 4
PTPN22: Protein tyrosine phosphatase-22
TSHR: Thyroid stimulating hormone receptor
TG: Thyroglobulin
RA: Rheumatoid arthritis
CD: Crohn’s disease
SSc: Systemic sclerosis
GWAS: Genome-wide association study
TRAb: Thyroid stimulating hormone receptor antibody
TPO-Ab: Anti-thyroid peroxidase antibody
Tg-Ab: Anti-thyroglobulin antibody
FNAB: Fine needle aspiration biopsies
